# Restoration of Hearing by Artificial Teeth

**Published:** 1854-04

**Authors:** Jas. S. Gilliams


*Extracts from a Thesis on the Restoration of Hearing by the Insertion of Artificial Teeth.* By Jas. S. Gilliams, M. D., D. D. S.

*Eustachian Tube.*—The principal object, for the fulfilment of which this tube exists, wherever there is a tympanum, appears to be the maintenance of the equilibrium between the air within the tympanum, and the external air, so as to prevent inordinate tension of the membrane tympani, which would be the case if too great or too little pressure was on either side, and the effect of which would be imperfection of hearing. It is not the increased or diminished density of the air, either side of the membrane, which is of the chief importance, but the tension of the membrane which is necessarily produced and which always interferes with the integrity of hearing. It is on this principle that the following cases may be explained:

In the year 1849, I received a message from Mrs. M., through the servant, with a set of teeth that required repairing, with the request that I would have them attended to as soon as possible, as Mrs. M. could not hear when without the teeth. This circumstance first attracted my attention, and when, at a subsequent time, she came to the office, I made more particular examination and found a great difference in her hearing in regard to what I said to her when her teeth were out, and when her teeth were in her mouth. About a year after, a lady applied for the insertion of an entire new set, she had never worn artificial teeth and her hearing was so much impaired that I was obliged to touch her chin and bellow in her ear when it was necessary that the mouth should be opened. When the teeth were inserted and the hand-glass presented to her, with the request that she should look at them, she suddenly exclaimed, "I hear everything you say—I can hear you perfectly well." The pleasure of thus recovering the power of hearing was so great, that she declared that this acquisition to be in her estimation of a great deal more importance to her than the ordinary advantages of the teeth. The power of hearing continued and she viewed the improvement in speaking, chewing, and the appearance of the mouth of secondary consideration. I have since witnessed the same phenomenon partially produced in several cases; one lady said she "could not listen without her artificial teeth." In two other cases something of the same kind was produced, but not in so marked a degree. These observations have led me to inquire whether any change could be induced in the orifice of eustachian tube by the excessive proximation of the jaws, such as to close or cover the orifice, or in any way impede the transmission of air through it. If we suppose at the same time, that deafness arose from want of balance between the air within the tympanum and that of the atmosphere, or that external deafness prevailed from causes affecting the membrane tympani, or from obstructions of the external meatus while the sensibility of the nerve was preserved, the causes of deafness might be more clearly understood.—*Dental News Letter.*

NOTE.—I have been trying to make more partial examinations, and obtained several heads for that purpose, but they were either too dry or injected with chloride of zinc.

